# Examining County-Level Associations between Federally Qualified Health Centers and Sexually Transmitted Infections: A Political Ecology of Health Framework

**DOI:** 10.3390/healthcare12030295

**Published:** 2024-01-24

**Authors:** Christopher Williams, Laura A. Skrip, Alexandrea S. Adams, Sten H. Vermund

**Affiliations:** 1 School of Natural and Social Sciences, State University of New York at Purchase College, Purchase, NY 10577, USA; 2 School of Public Health, College of Health Sciences, University of Liberia, Monrovia 1000-10, Liberia; skripla@ul.edu.lr; 3 Quantitative-Data for Decision-Making Lab, Monrovia 1000-10, Liberia; 4 School of Medicine, Wake Forest University, Winston Salem, NC 27157, USA; adamsalexs14@gmail.com; 5 School of Public Health, Yale University, New Haven, CT 06510, USA; sten.vermund@yale.edu; 6 School of Medicine, Yale University, New Haven, CT 06510, USA

**Keywords:** sexually transmitted infections, federally qualified health centers, Medicaid expansion, social vulnerability, metropolitan, political ecology of health

## Abstract

Federally Qualified Health Centers (FQHCs) are the largest providers of healthcare for sexually transmitted infections (STIs) in medically underserved communities in the United States (US). Through the Affordable Care Act (ACA), FQHCs have grown in number, but the impact of this growth on STIs is poorly understood. This ecological study seeks to quantify the association between FQHCs and STI prevalence in all US counties. Variables were described utilizing medians and interquartile ranges, and distributions were compared using Kruskal-Wallis tests. Median rates of chlamydia in counties with high, low, and no FQHCs were 370.3, 422.6, and 242.1 cases per 100,000 population, respectively. Gonorrhea rates were 101.9, 119.7, and 49.9 cases per 100,000 population, respectively. Multivariable linear regression models, adjusted for structural and place-based characteristics (i.e., Medicaid expansion, social vulnerability, metropolitan status, and region), were used to examine county-level associations between FQHCs and STIs. Compared to counties with no FQHCs, counties with a high number of FQHCs had chlamydia rates that were an average of 68.6 per 100,000 population higher (β = 68.6, 95% CI: 45.0, 92.3) and gonorrhea rates that were an average of 25.2 per 100,000 population higher (β = 25.2, 95% CI: 13.2, 37.2). When controlled for salient factors associated with STI risks, greater FQHC availability was associated with greater diagnosis and treatment of STIs. These findings provide empirical support for the utility of a political ecology of health framework and the critical role of FQHCs in confronting the STI epidemic in the US.

## 1. Introduction

Over the past decade, sexually transmitted infection (STI) rates have increased substantially and rank among the most pressing public health concerns [1,2,3]. In the United States (US), STI rates are disproportionately high relative to the rates in other high-income nations [4]. It is estimated that one in five individuals in the US has an STI on any given day [1]. In 2021, 1,644,416 cases of Chlamydia trachomatis (CT), the most prevalent STI in the US, were reported to the Centers for Disease Control and Prevention (CDC). This figure corresponds to a rate of 495.5 per 100,000 population and a 3.9% increase from 2020 [1]. For the second most common notifiable STI, Neisseria gonorrhoeae (NG), 710,151 cases were reported, corresponding to a rate of 214.0 cases per 100,000 population and a 4.6% increase from 2020 to 2021 [1]. Furthermore, increasing STI incidence is exacerbated by global antimicrobial-resistant gonorrhea, a growing public health threat in the US [5].

Access to prompt healthcare is associated with early diagnosis, lower community infectivity, and effective curative treatment [6]. Conversely, inadequate access to STI care is associated with a risk of reinfection, onward transmission, and HIV exposure [7,8]. Women with untreated infections are at risk for pregnancy complications, congenital and neonatal infections, and infertility [9,10]. Men with untreated infections are at increased risk for urethritis, epididymitis, and proctitis [1]. In addition to the physical impact of STIs, econometric data estimate the direct medical cost burden for CT and NG to be $1.1 billion (2019 US dollars) annually, including costs of treating infection and sequelae costs of untreated or inadequately treated infection [11]. Throughout the US, governments (i.e., local, state, and federal) coordinate funding for programs that deliver STI screening, prevention, and treatment services; however, these efforts are often fragmented, vary widely across jurisdictions, and can produce substantial safety-net service gaps [12].

### 1.1. Federal and State Provisions for Sexual Healthcare

Longstanding structural factors can impede access to STI testing and treatment and are important drivers of health disparities. To mitigate the impact of such disparities, the Affordable Care Act (ACA) established the Community Health Fund, which led to a dramatic increase in funding and growth of federally qualified health centers (FQHCs) [13]. FQHCs are healthcare providers that are federally mandated to provide primary care and treatment for communicable diseases to patients regardless of ability to pay, making them a critical access point for the underinsured or uninsured [14,15]. The ACA also expanded Medicaid, a government entitlement program that provides health insurance to more than 30 million people living at or near the federal poverty level. A majority of Medicaid beneficiaries receive healthcare from FQHCs [13]. The ACA legislation has made Medicaid the largest single payer of STI claims and the largest source of revenue for FQHCs [16,17,18]. Medicaid expansion is linked to increased access to care and significant reductions in STI morbidity and mortality—especially among racial/ethnic minorities and residents of poorer counties [19]. Although a federal program, Medicaid is partially funded and primarily managed by state governments. Through a type of cooperative federalism, federal policymakers establish national policies that are operationalized through funding to states [12]. However, states maintain the authority to determine eligibility and benefits, resulting in wide variability in FQHC numbers and funding through disparate combinations of Medicaid, Medicare (a federal insurance entitlement program for the elderly and disabled), private insurance, self-pay, and state, local, and federal grants [17]. As of early 2024, 10 states have refused federal funding under the ACA to expand Medicaid coverage—Alabama, Florida, Georgia, Kansas, Mississippi, South Carolina, Tennessee, Texas, Wisconsin, and Wyoming—and have over 2 million people whose incomes would make them Medicaid-eligible if their states chose to participate in ACA-supported Medicaid expansion.

FQHCs are embedded within communities and are considered important in reducing morbidity; however, research examining the impact of these safety-net providers on STI rates has produced mixed results. One study suggested an association between the number of FQHCs and lower STI indices, though the association was not significant after adjusting for population size [20]. Standards of care at FQHCs are also important considerations. In a large Midwestern FQHC system, CT and NG screening was only provided upon patients’ request or when symptoms were present, despite high community infection rates [21]. In another study of urban FQHCs, patients were not routinely asked about sexual health concerns, regardless of their willingness to engage with providers [22]. In a large multi-site review of FQHC electronic health records, screening for hepatitis C infection was infrequent despite high local community injection drug use rates [23]. The variability in service offerings is illustrated in another FQHC study reporting that a majority of patients were tested for CT and NG with treatment of positive cases, but few were retested, and screening and testing for HIV or syphilis did not meet CDC standard of care guidelines [24]. Nevertheless, research suggests that FQHCs may play a critical role in addressing entrenched safety-net service gaps in the ACA and Medicaid expansion era when the underserved has had access to a wider range of service providers [6,25]. Prior studies point to the need for an integrated conceptual model that considers salient determinants of health, healthcare infrastructure, quality of care, and government funding in the control and elimination of STIs in the US.

### 1.2. A Political Ecology of Health Model

A review of the extant literature indicates that key drivers of the upsurge in CT and NG infections are multifactorial though most studies emphasize person-level factors (e.g., individual risk behaviors) without consideration of structural determinants [2]. Utilization of broad-scale analytical and conceptual approaches can consider proximal and distal structural drivers simultaneously. One such approach is Mayer’s political ecology of health framework that integrates disparate social, economic, and political determinants of disease into one model [26,27]. The political ecology of health model is particularly relevant given that STI is driven not only by exposure to opportunistic biological pathogens but also by a myriad of societal determinants [2,12]. Arguably, fiscally restrictive health policies and laws can exacerbate societal determinants and can figure heavily into population-based STI morbidity and mortality [28,29,30]. A political ecology model provides a comprehensive analytical framework with which to examine STIs and the extent to which these health conditions are influenced by the allocation of government and public health resources and other structural factors.

A political ecology model is also anchored on the recognition that a pathogen and host must have mutual contact at the same place and time. As such, place-based studies are especially important in the US, where health disparities, including STIs, often vary substantially by geographic location. For example, health disparities are most pronounced in southern states, where 38% of the nation’s population resides [31]. The South, made up of rural areas as well as densely populated urban areas, has the highest rates of all STIs [29,30,31,32]. Drivers of STIs in rural areas can result from fewer service locations, shortages in providers, cultural differences, and stigma [16,30,33]. Disadvantaged geographic location is exacerbated in urban settings where there are often lower levels of educational attainment, poverty, long clinic waiting times, low-risk perception, and provider-patient mistrust [16,34,35]. Political ecology models can serve as a fruitful explanatory framework for infectious and noncommunicable diseases. One ethnographic analysis considered social determinants of HIV and tuberculosis infections by placing them within a political ecology model that weighs poverty, unemployment, limited healthcare access, place-based factors, and comorbidities of people who are immunodeficient [36]. Another ethnographic study considered the utility of a political ecology framework when examining vulnerability to metabolic diseases, specifically the interplay between physiological and social aspects of obesity. The most effective approaches for understanding obesity were asserted to be those that draw together political, economic, and biomedical perspectives [37]. Combined, this growing body of work underscores that there is ample theoretical consideration for the utility of political ecology models in disease etiology, but there remains a dearth of empirical validation of this conceptually expansive framework.

### 1.3. Purpose of Study

During an era of declining public funding for STI control and elimination, it is critical to investigate healthcare access especially among communities that face longstanding barriers to care and elevated morbidity and mortality. We examined the influence of Medicaid expansion, social vulnerability, place-based factors (e.g., metropolitanism) and FQHCs on STI outcomes. To assess county-level associations between FQHCs and STI morbidity, we proposed several integrated hypotheses. First, because Medicaid expansion increases access to primary healthcare, we hypothesized that counties located in states where Medicaid had been expanded are likely to offer more diagnostic capacity and treatment [16,18,19]. Because high social vulnerability (e.g., poverty, low educational attainment, poor housing conditions) compared to low social vulnerability has been linked to increased STI rates [25,28,29], we predicted similar associations in the present study. We also hypothesized that urban (metropolitan) relative to nonurban counties would likely have higher STI rates due, in part, to unbalanced healthcare provider-to-patient ratios, and entrenched provider-patient mistrust among racial/ethnic minority groups [3]. Across a consistent body of research, southern states report higher STI rates relative to other regions [33,35]. We predicted to observe similarly high rates. Finally, there is limited research on the impact of FQHCs on STI outcomes. The available research often involves smaller-scale geographic regions and strictly defined subpopulations [20,21,22,23,24]. Of the studies available, none of them has considered the impact of these safety-net providers in the context of an ecological health model. In our population-based analysis, we hypothesized that counties with more FQHCs per capita would be associated with greater diagnosis and treatment available to its residents. However, the higher risk communities might mask such a putative benefit without consideration of potentially confounding factors. Given the heterogeneity in FQHC availability between and within states, a county-level analysis of STI morbidity is timely and has important public health relevance.

## 2. Materials and Methods

Using publicly available sources, we extracted US county-level data, including: (1) STI rates; (2) number of FQHCs; (3) Medicaid expansion status (given that not all states have participated in the expansion of services through the ACA); (4) social vulnerability; (5) metropolitan status; and (6) US Census region. All data were linked by county Federal Information Process Standard (FIPS) codes [38]. The dataset was created in December of 2022 with measures ranging from 2017 to 2019. STI rates for 2019 were selected because, at the time of data extraction, 2019 was the most current data available. Dates for predictor variables were selected to ensure at least one year between each predictor variable and 2019 outcomes. Statistical analyses were finalized in September 2023.

### 2.1. Outcome Measures

Aggregated rates of chlamydia and gonorrhea (all ages and sexes) were selected for their relatively high burden on the US population and because they are systematically reported by counties based on infection positivity—defined as a positive nucleic acid amplification test (NAAT) at any anatomic site. Annual CT and NG 2019 prevalence data (cases per 100,000 population) were available and extracted for 3138 counties and county equivalents (99% of 3144 total) in all 50 states and the District of Columbia from the CDC National Notifiable Disease Surveillance System database [39].

### 2.2. Covariates

#### 2.2.1. Federally Qualified Health Centers

FQHC sites and locations were extracted from the 2018 Health Resources and Health Administration database [40]. We did not include FQHCs that were designated ‘administrative only’ or that had other nonprimary medical care designations (e.g., domestic violence centers) because our focus was to examine FQHCs that deliver primary care including sexual health. Using those inclusion parameters, we identified 3142 eligible FQHCs. The median number of county FQHCs was 3.53 per 100,000 population. Counties with a total number of FQHCs above the median were categorized as ‘high’, and those with a total number below the median were categorized as ‘low’. A third category labeled ‘none’ was created for counties with no FQHCs. The distribution of FQHC frequency categories included ‘high’ (*n* = 1391), ‘low’ (*n* = 760), and ‘none’ (*n* = 991). This three-level category permitted observation of trends (e.g., dose-response), comparison of noncontiguous groups (e.g., high vs. low), and ease of interpretation of coefficients (e.g., ‘change in STI rates per category’ was used rather than ‘per unit change’).

#### 2.2.2. Medicaid Expansion Status

The Affordable Care Act (ACA) was signed into law in 2010 and began to be implemented in 2014. The ACA increased Medicaid funding to states so that uninsured and underinsured residents could have access to basic healthcare. Data regarding states’ status on Medicaid expansion were obtained from a Centers for Medicare and Medicaid Services database [41]. To coincide with other covariates and the STI outcomes, we classified this state-level variable for each county based on whether Medicaid was: (1) expanded before 2017; (2) expanded during 2017 or after; or (3) not expanded as of 2019, hereafter referred to as ‘never’ during our time period of study.

#### 2.2.3. Social Vulnerability

Social Vulnerability Index (SVI) data were obtained from the 2018 CDC/Agency for Toxic Substances and Disease Registry [32,42]. The CDC calculated SVI based on county-level estimates of 15 social factors (e.g., poverty, adults without high school diplomas, percent of households with geriatric or pediatric residents, percent of minorities, percent of crowded homes, and percent of homes without vehicles available). These 15 factors were grouped into four themes: (1) socioeconomic status; (2) household composition and disability; (3) minority status; and (4) housing type and transportation. For each US county and for each of the 15 factors, the CDC calculated an SVI score ranging 0 to 1, with higher values indicating greater vulnerability. Specifically, each county’s score reflects the percentile ranking of the county in terms of its vulnerability on that score, such that the score is the proportion of all counties nationally that are equally or less vulnerable. For each theme, the percentile ranks of the factors in that theme are summed. That is, the thematic score reflects the ranking of the summed factor percentile ranks. In addition to theme scores, the CDC calculated an overall SVI score (a sum across all 4 thematic scores), also ranging from 0 to 1. We used the overall SVI score for each county and conducted our analysis with tertiles: low, 0.00–0.33; medium, 0.34–0.66; and high, 0.67–1.00 [28,43].

#### 2.2.4. Metropolitan Status

Urbanicity of counties was defined using the six-level classification scheme of the National Center for Health Statistics [44]. There were four levels for metropolitan urban counties (large central, large fringe, medium, and small) and two levels for nonmetropolitan rural counties (micropolitan and noncore). We examined metropolitan status as a dichotomous variable (metropolitan urban vs. nonmetropolitan rural).

#### 2.2.5. US Census Regions

Using Census data, we condensed nine subregions (New England Middle Atlantic, East North Central, West North Central, South Atlantic, East South Central, West South Central, Mountain, Pacific) to four regions (Northeast, Midwest, South, West) [45].

### 2.3. Statistical Analysis

We selected US county as the unit of analysis because of its availability for the primary exposure (FQHCs) and outcome (STIs). First, descriptive statistics (i.e., median and interquartile range [IQR]) were computed and statistical significance was assessed with Kruskal-Wallis tests. Secondly, using unadjusted linear regression models, we examined the association between FQHCs and median STI rates (per 100,000 population). Thirdly, we re-examined the association between FQHCs and STI rates, and adjusted for potential confounders, including Medicaid expansion, social vulnerability, metropolitan status, and US Census region. Based on data availability and the assumption that county 2019 STI rates would have been influenced by potential structural predictors, we used the number of FQHCs and SVI from 2018, and Medicaid expansion status from 2017. Fourthly, we examined differences in the outcome (median rates) across exposure (FQHC levels) using stratified analyses. Finally, we ran four sensitivity analyses to assess the robustness of study findings and model estimations as follows: (1) because CT and NG rates were not normally distributed (i.e., they were right-skewed), we re-ran the primary linear regression models (FQHCs with all covariates) using log-transformed rates; (2) we re-ran models with continuous rather than categorical measures; (3) we re-ran models with an index of outcomes (combined CT and NG rates per 100,000 population); and (4) we restricted the analytic sample to counties with 10% or greater of the population living at or below the federal poverty level. Significance was defined as *p* < 0.05 using a two-tailed alpha. All analyses were conducted using IBM SPSS Statistics v 20 [46].

### 2.4. Ethics

All data were available in aggregate in the public domain and did not include any contact with human participants. Hence, this study was determined to be exempt by the Institutional Review Board at the State University of New York at Purchase College.

## 3. Results

First, we sorted the top 10 counties from the most to least number of FQHCs. The highest numbers of FQHCs included Los Angeles County, California (587), Cook County, Illinois (247), Miami-Dade County, Florida (237), San Diego County, California (204), Bronx County, New York (161), Alameda County, California (155), Kings County, New York (144), King County, Washington (124), New York County, New York (122), and Hartford County, Connecticut (109). We also sorted counties from the most to least number of FQHCs per 100,000. This list revealed that none of the counties included on the first top 10 list was among the FQHC per 100,000 list. Finally, we observed that the majority of counties without any FQHCs were found in the South (502) and Midwest (340) regions.

In 2019, median county CT and NG rates were 333.2 (IQR, 219–507) and 83.9 (IQR, 40–170) cases per 100,000 population, respectively. Median rates of STIs by FQHCs levels, Medicaid expansion status, social vulnerability index, metropolitan status, and Census regions are shown in Table 1.

Bivariable analyses revealed that CT and NG were associated with greater FQHC numbers, state non-participation in Medicaid expansion, urban metropolitan status, and Southern region. In separate unadjusted linear regression models, greater numbers of FQHCs were significantly associated with higher CT and NG rates. For example, compared to counties with no FQHCs, counties with a high number of FQHCs had CT rates that were an average 179.0 per 100,000 population higher (β = 179.0, 95% CI: 155.8, 202.1; *p* < 0.001) and NG rates that were an average 78.6 per 100,000 higher (β = 78.6, 95% CI: 66.9, 90.3; *p* < 0.001). Likewise, compared to counties with no FQHCs, counties with a low number of FQHCs had CT rates that were an average 192.4 per 100,000 population higher (β = 192.4, 95% CI: 170.4, 214.5; *p* < 0.001) and NG rates that were an average 79.9 per 100,000 population higher (β = 79.9, 95% CI: 68.5, 91.3; *p* < 0.001).

In adjusted multiple linear regression models (adjusted for Medicaid expansion, SVI, metropolitan status, and Census region, Table 2), higher FQHC levels remained significantly associated with higher CT and NG rates. For example, compared to counties with no FQHCs, counties with a high number of FQHCs had CT rates that were an average 68.6 per 100,000 population (β = 68.6, 95% CI: 45.0, 92.3; *p* < 0.001) and NG rates that were an average 25.2 per 100,000 (β = 25.2, 95% CI: 13.2, 37.2; *p* < 0.001).

Likewise, compared to counties with no FQHCs, counties with a low number of FQHCs had CT rates that were an average 122.4 per 100,000 population higher (β = 122.4, 95% CI: 99.3, 145.5; *p* < 0.001) and NG rates that were on average 46.2 per 100,000 population higher (β = 46.2, 95% CI: 34.3, 58.2; *p* < 0.001). Medicaid expansion status, social vulnerability index, metropolitan status, and Census region were also associated with CT and NG. Multicollinearity checks were within the recommended limit of 10. The highest variance inflation factor was 1.54 (mean = 1.35) for all infection models.

We also examined differences in STI outcomes (median rates) across exposure levels (FQHCs) by conducting stratified analyses (Table 3).

Overall, higher CT and NG rates were observed in counties with high compared to low numbers of FQHCs, when key co-factors were considered in multivariable models. For all Medicaid levels, higher STI rates were observed in counties with high numbers of FQHCs compared to counties without any FQHCs. For all levels of social vulnerability, higher STI rates were observed in counties with high numbers of FQHCs compared to counties without any FQHCs. Across both levels of metropolitan status (urban and rural), higher STI rates were observed in counties with high numbers of FQHCs compared to counties with no FQHCs. In all subregions of the US, higher STI rates were observed in counties with high numbers of FQHCs compared to those without any FQHCs. 

Sensitivity analyses (shown in Supplemental Table S1) indicated that the main findings were robust to model assumptions and measurement approaches (e.g., categorization of variables based on tertiles) <see supplemental material for Table S1>. The first sensitivity analysis used linear regression models with log-transformed chlamydia and gonorrhea rates as the outcome variables and yielded quantitatively similar results in terms of associations with FQHCs, goodness of fit statistics, adjusted *R*^2^ values, and residual diagnostics; as such, the untransformed estimates are presented for ease of interpretation. Three additional models using: (a) continuous, rather than categorical covariates; (b) combined outcomes (CT and NG); and (c) a higher poverty subsample were implemented as part of the sensitivity analysis. These analyses also yielded results similar in terms of direction, magnitude, and significance to the primary analysis.

## 4. Discussion

Using a population-based study design, we examined the impact of FQHCs on STIs in the US. Overall, we found that higher chlamydia and gonorrhea were observed in counties with more FQHCs compared to those with lower FQHCs, controlling for Medicaid expansion, social vulnerability, metropolitan status, and region of the country. Our ecological study suggests that FQHCs are needed for sexual healthcare among medically underserved populations as they are disproportionately serving at-risk populations. Although safety-net providers have been examined for their role in reducing STI morbidity and mortality; to the best of our knowledge, this study is the first to examine the association of FQHC rate (quantity per county) and STI prevalence utilizing a county-level study design with analytical consideration of nascent state policy decisions and other structural factors.

Controlling for covariates, our study found that low or high numbers of FQHCs in a county were associated with higher STI rates than no FQHCs in a county—suggesting that greater FQHC availability should be linked to more extensive STI screening and diagnosis and treatment. This finding is especially important as patients’ use of FQHCs often varies as a function of local FQHC capacity and availability of non-FQHC providers [13]. Indeed, research suggests that many FQHCs are underfunded and cannot hire additional clinical staff or expand geographical reach and service offerings [47]. Furthermore, studies have shown that FQHC growth has tended to occur in pre-existing service markets, urban areas, and locales with lower poverty rates, suggesting the influence of market-driven factors [12,48]. Although FQHCs face a number of challenges, a body of research indicates their positive impact on STI care [13,20,21]. In this study, we found FQHC availability to be associated with higher rates of bacterial STIs, and this association persisted even when considering Medicaid expansion—an important driver of health outcomes. This finding is notable given that Medicaid expansion has increased access to traditional safety-net services as well as private providers. Given that FQHCs target lower income communities, one would predict higher STI rates in such communities that face greater structural barriers to healthcare access. Our study findings suggest that convenient primary care through FQHCs must include earlier STI detection, care, and prevention of transmission. 

Access to screening, early diagnosis, and effective curative treatment are essential for STI control and elimination. However, patient populations in the most vulnerable US counties often contend with a myriad of barriers that adversely impact their health status. A decade after the ACA went into effect, the FQHC program remains focused on reducing long-standing health disparities [13,14]. A compelling body of research suggests that the primary drivers of these disparities are racial discrimination [29,49,50], poverty [30,49,50], and geographic location [26]. In this study, we found that these factors, captured in part by social vulnerability, were more pronounced in areas with protracted legislative hesitancy toward Medicaid expansion. At its core, states’ resistance toward Medicaid expansion—especially in the South—appears more ideologically than fiscally driven, as the federal government’s original commitment to expansion covered the entire cost of insuring new Medicaid patients for the first 3 years of implementation of the ACA and 90% thereafter [51]. The ideological drivers of Medicaid resistance are further illustrated by anti-immigrant policies and legislative initiatives that act as barriers to seeking safety-net STI care among socially and economically marginalized groups [52,53,54]. The net effect of restrictive health policy decision making may be important to consider when examining STI morbidity [13,14]. In this study, we observed in high-social vulnerability counties in the deep South, STI rates were nearly three times higher than in low-vulnerability counties—a finding consistent with other epidemiologic studies [28,29]. We also found significantly higher STI rates in metropolitan compared to nonmetropolitan counties. Combined, these factors may have important predictive value for STIs morbidity across regions of the country with disparate fiscal and health policy approaches, patient populations, and epidemiological patterns.

The factors contributing to sustained transmission of STIs are multifactorial and thus, the most comprehensive analyses are likely to include economic, political, and social contextual factors [51]. Recognizing the complexity of health determinants, we utilized a political ecology model to analyze and interpret our findings. A primary tenet of this model is the consideration of large-scale influences that shape the health structures of local environments. In this regard, our finding of increased STI morbidity in socially vulnerable counties may be attributable to policies and laws that limit the allocation of healthcare resources. Our finding that the association between FQHC numbers, states’ positions on Medicaid expansion, and STI prevalence provides empirical support for the influence of economic and political drivers of healthcare access. This structural approach helps to clarify how changes in health policies, laws, and infrastructure impact STI morbidity.

Nearly half of all FQHC patients are Medicaid beneficiaries [14]. However, even after the ACA implementation, there were over 27 million people living below the federal poverty line who remain uninsured because their states had not expanded the Medicaid entitlement program in our study period—a consequential structural intervention [55,56]. Furthermore, even Medicaid beneficiaries must comply with byzantine requirements to prove eligibility or face disruption or complete termination of healthcare coverage [57]. This study suggests that social vulnerability and restrictive funding decisions may operate synergistically. These determinants in and of themselves reflect entrenched legislative and policy choices that may not be in the best interest of public health. As such, examining a range of local, state, and federal health policy decision making is essential for the most rigorous evaluations of the US STI epidemic.

### Limitations and Strengths

This study includes a comprehensive analysis of the association of FQHC availability and chlamydia and gonorrhea morbidity and is among the first to examine these factors across all US counties. However, there are several limitations that should be considered when interpreting the present results. First, we acknowledge that county STI rates are likely to be undercounts due in part to variation in health-care-seeking behavior among those infected. As a result, low case rates may indicate either low infection, undiagnosed, or unreported infection. In this study, we believe there may be substantial underreporting in counties without FQHCs due either to lower diagnostic capacity or, in some cases, because residents of these counties do not use safety-net services for STI care. If underreporting is a valid assumption, our findings may differ from the true relationships. Secondly, counties are mandated by the federal government to report STI rates to the CDC. However, there is wide variability in the quality of these reports. Given this, STI rates should be considered as trends, subjected to consistent bias, but may be valid nevertheless. A third limitation is that our analyses are based on the assumption that a greater number of FQHCs in a county represents an increase in the number of patients effectively treated. This assumption may not be valid given the potential for wide variation in the quality of clinical practice [58]. Fourthly, we acknowledge the complexity of comparing STI outcomes across all FQHC levels, high, low, as well as none, since the absence of FQHCs in a county may reflect a gap in STI-related services—availability and/or utilization—if other safety-net healthcare services are unavailable (STI services are typically included in FQHCs). We tested this assumption in our sensitivity analysis. Specifically, we re-ran a linear regression model that restricted the sample to only counties with poverty rates of 10% or higher. This analysis revealed that associations between FQHCs and STI rates were similar in magnitude to findings from analyses including all counties—suggesting that the association between FQHC availability and STI outcomes may reflect complex dynamics other than those related to poverty or need for safety-net healthcare services alone. However, given some uncertainty about characteristics of counties without any FQHC, we have presented our findings with caution. Finally, the ecological nature of this study precludes us from establishing the most direct effect between FQHC availability and STIs because we did not collect data on personal factors (e.g., risk behaviors, comorbidities). Future studies could use patient-level data in an interrupted time series design to explore STI rates before and after FQHC services become available.

Notwithstanding these limitations, this study is among the first to evaluate the association between FQHCs and two common and curable STIs (chlamydia and gonorrhea) across US counties that are heterogeneous in their provision of healthcare services. Furthermore, our findings are supported by a number of methodological strengths. First, we used a population-based study design while considering the influence of Medicaid expansion—a large-scale federal legislative driver of healthcare access. Secondly, we also considered in our models the influence of important place-based factors (urbanism and region of the county). Thirdly, we present findings based on a diversity of FQHCs in all US counties rather than a single state or FQHC system. Fourthly, we used a rigorous data analytic strategy that included observations of trends, comparison of noncontiguous groups, and sensitivity testing.

## 5. Conclusions

There has been a global resurgence of STIs. In the US, this resurgence is most pronounced in medically underserved communities that also bear the burden of the most restrictive allocation of healthcare resources. Additionally, the spread of antimicrobial resistance has heightened concern about limited treatment options for STIs. In light of these factors, FQHCs will be an increasingly important component of the primary healthcare system. Our study adds to the body of evidence regarding the importance of the growth of FQHCs following implementation of Medicaid expansion under the ACA. The present findings suggest that the availability of FQHCs is associated with higher CT and NG rates, indicating the importance of greater utilization of STI diagnosis and treatment services and, thus, lowering infection rates. The relationship between FQHCs and STIs observed in this study suggests that FQHC safety-net care and public health infrastructure are essential in medically underserved communities. Finally, this work provides empirical support for the utility of a political ecology model of health that integrates the influence of various county, state, and federal contextual factors that sustain STI epidemics.

## Figures and Tables

**Table 1:** Bivariable associations between county-level median annual chlamydia and gonorrhea rates per 100,000 population in 2019 and covariates *.

	Chlamydia	Gonorrhea
	per 100,000 population	per 100,000 population
Overall	333.2	83.9
Federally Qualified Health Centers ^‡^		
High	370.3	101.9
Low	422.6	119.7
None	242.1	49.8
Medicaid Expansion		
Expanded before 2017	311.2	70.0
Expanded in 2017 or after	299.0	70.0
Never	391.6	115.6
Social Vulnerability ^‡‡^		
Low	244.4	47.1
Medium	323.5	82.8
High	509.9	172.4
Metropolitan Status		
Metro	398.7	110.6
Non-metro	293.8	64.7
Census Region		
Northeast	294.6	44.3
Midwest	268.3	59.2
South	415.1	129.3
West	337.1	62.1

* All covariates were significantly associated with chlamydia and gonorrhea by Kruskal-Wallis test. (*p* < 0.001) except for Metropolitan Status and FQHCs rates (*p* < 0.01). ^‡^ High and Low FQHCs were above and below the median split of 3.53 per 100,000 residents in county, and None represents counties having no FQHCs. ^‡‡^ Social Vulnerability Index (SVI) measures: (1) socioeconomic status; (2) household composition and disability; (3) minority status and language; and (4) housing and transportation.

**Table 2:** Adjusted ^†^ beta coefficients and 95% confidence intervals for association between Federally Qualified Health Centers (FQHC) in 2018 and chlamydia and gonorrhea rates in 2019 from linear regression.

	Chlamydia	Gonorrhea
	Beta (95% CI)	Beta (95% CI)
Federally Qualified Health Center ^‡^		
High v. None	68.6 (45.0, 92.3) ***	25.2 (13.2, 37.2) ***
Low v. None	122.4 (99.3, 145.5) ***	46.2 (34.3, 58.2) ***
Medicaid Expansion		
Expanded before 2017 v. Never	21.5 (0.23, 42.8) *	20.5 (10.2, 30.8) ***
Expanded before 2017 or after v. Never	−4.5 (−33.6, 24.50)	−8.7 (−23.7, 6.4)
Social Vulnerability ^‡‡^		
High v. Low	320.4 (293.4, 347.3) ***	164.7 (151.0, 178.4) ***
Medium v. Low	105.0 (90.2, 119.8) ***	49.2 (42.1, 56.3) ***
Metropolitan Status		
Metro v. Non-metro	125.5 (106.4, 144.3) ***	54.5 (44.9, 64.2) ***
Census Region		
Midwest v. Northeast	38.0 (7.3, 68.7) **	60.0 (41.5, 78.5) ***
South v. Northeast	22.5 (−23.3, 68.3)	39.7 (18.1, 61.3) ***
West v. Northeast	24.2 (−23.7, 72.1)	29.5 (7.3, 51.8) **

* *p* < 0.05; ** *p* < 0.01; *** *p* < 0.001. ^†^ Adjusted models included measures of Medicaid status, social vulnerability, metropolitan status, and Census region. ^‡^ High and Low FQHCs were above and below the median split of 3.53 per 100,000 residents in county, and None represents counties having no FQHCs. ^‡‡^ Social Vulnerability Index (SVI) measures: (1) socioeconomic status; (2) household composition and disability; (3) minority status and language, and (4) housing and transportation.

**Table 3:** Median chlamydia and gonorrhea rates in 2019 per 100,000 population stratified by FQHC level.

	Federally Qualified Health Center ^‡^			
	High	Low	None	*p*-Value
	per 100,000 population	per 100,000 population	per 100,000 population	
Chlamydia				
Medicaid Expansion				
Expanded before 2017	340.7	386.9	240.5	<0.001
Expanded in 2017 or after	335.4	435.9	226.6	<0.001
Never	454.1	476.8	253.7	<0.001
Social Vulnerability ^‡‡^				
Low	256.7	329.5	216.3	<0.001
Medium	315.7	447.4	257.4	<0.001
High	518.7	606.9	397.1	<0.001
Metropolitan Status				
Metro	436.5	441.7	268.3	<0.001
Non-metro	337.4	377.2	233.3	<0.001
Census Region				
Northeast	289.8	306.8	256.7	<0.05
Midwest	297.7	383.5	224.8	<0.001
South	435.8	490.7	331.9	<0.001
West	380.4	431.9	165.5	<0.001
Gonorrhea				
Medicaid Expansion				
Expanded before 2017	83.3	95.2	44.8	<0.001
Expanded in 2017 or after	94.2	132.8	43.7	<0.001
Never	143.6	155.8	62.9	<0.001
Social Vulnerability ^‡‡^				
Low	48.6	71.1	38.2	<0.001
Medium	83.2	132.1	55.2	<0.001
High	172.3	235.2	128.0	<0.001
Metropolitan Status				
Metro	130.7	130.2	69.9	<0.001
Non-metro	88.0	94.5	43.1	<0.001
Census Region				
Northeast	40.0	50.8	27.2	<0.05
Midwest	80.1	102.0	41.1	<0.001
South	137.2	167.8	90.4	<0.001
West	87.2	90.4	18.1	<0.001

^‡^ High and Low FQHCs were above and below the median split of 3.53 per 100,000 residents in county, and None represents counties having no FQHCs. ^‡‡^ Social Vulnerability Index (SVI) measures: (1) socioeconomic status; (2) household composition and disability; and (3) minority status and language; and (4) housing and transportation.

## Data Availability

All data were extracted from publicly available data sources described in the Methods and Materials section and linked in the Reference section.

## Supplementary Materials

The following supporting information can be downloaded at https://www.mdpi.com/article/10.3390/healthcare12030295/s1, Table S1: Sensitivity Analysis: Results (beta coefficients and 95% CIs) for association between FQHC rates and STI (chlamydia and gonorrhea) from fully adjusted models.
