# Supplementary material for: Examining County-Level Associations between Federally Qualified Health Centers and Sexually Transmitted Infections: A Political Ecology of Health Framework
Source: Healthcare (Basel). 2024 Jan 24;12(3):295. doi: 10.3390/healthcare12030295 (PMC10855137; doi:10.3390/healthcare12030295)
Supplement: Supplementary file 1 [file healthcare-12-00295-s001.zip › healthcare-2768984-supplementary.pdf]

Table S1. Sensitivity Analysis.

**Results (Beta Coefficients and 95% CIs) for Association between FQHC Rates and STI (Chlamydia and Gonorrhea) from Fully Adjusted Model**			
Primary Analysis			
Linear Regression<br>STI: continuous<br>FQHC: median split<br>Covariates: dichotomous or tertiles			
	Chlamydia		
		High v No FQHCs	68.6 (45.0, 92.3)***<br>Model diagnostics: F stat p-value < 0.001, adjusted R^2 = 0.244, mean residual = 0.
		Low v No FQHCs	122.4 (99.3, 145.5) ***<br>Model diagnostics: F stat p-value < 0.001, adjusted R^2 = 0.344, mean residual = 0.
	Gonorrhea		
		High v No FQHCs	25.2 (13.2, 37.2) ***<br>Model diagnostics: F stat p-value < 0.001, adjusted R^2 = 0.218, mean residual = 0.
		Low v No FQHCs	46.3 (34.3, 58.2) ***<br>Model diagnostics: F stat p-value < 0.001, adjusted R^2 = 0.314, mean residual = 0.
Secondary Analysis 1			
Linear Regression<br>STI: log transformed<br>FQHC: median split<br>Covariates: dichotomous or tertiles			
	Chlamydia		
		High v No FQHCs	0.09 (0.06, 0.11) ***<br>Model diagnostics: F stat p-value < 0.001, adjusted R^2 = 0.300, mean residual = 0.
		Low v No FQHCs	0.16 (0.14, 0.19) ***<br>Model diagnostics: F stat p-value < 0.001, adjusted R^2 = 0.346, mean residual = 0.
	Gonorrhea		
		High v No FQHCs	0.06 (0.03, 0.09) ***<br>Model diagnostics: F stat p-value < 0.01, adjusted R^2 = 0.314, mean residual = 0.
		Low v No FQHCs	−0.16 (0.12, 0.20) ***<br>Model diagnostics: F stat p-value < 0.001, adjusted R^2 = 0.359, mean residual = 0.
Secondary Analysis 2			
Linear Regression<br>STI: continuous<br>FQHC: continuous<br>Covariates: continuous			
	Chlamydia		2.45 (1.85, 3.12) ***
	Gonorrhea		0.45 (0.79, 1.43) ***
Secondary Analysis 3			
Linear Regression<br>STI: Combined			

FQHC: median spilt<br>Covariates: dichotomous or tertiles		
	High v No FQHCs	46.9 (30.0, 63.8) ***<br>Model diagnostics: F stat p-value < 0.001, adjusted R^2 = 0.254, mean residual = 0.
	Low v No FQHCs	84.3 (67.7, 100.9) ***<br>Model diagnostics: F stat p-value < 0.001, adjusted R^2 = 0.356, mean residual = 0.
Secondary Analysis 4		
Linear Regression<br>STI: continuous<br>FQHC: median split<br>Covariates: dichotomous or tertiles<br>Sample restricted to county poverty rates $\geq$ 10%		
Chlamydia		
	High v No FQHCs	72.0 (43.6, 100.3) ***<br>Model diagnostics: F stat p-value < 0.001, adjusted R^2 = 0.208, mean residual = 0.
	Low v No FQHCs	129.3 (99.6, 159.0) ***<br>Model diagnostics: F stat p-value < 0.001, adjusted R^2 = 0.315, mean residual = 0.
Gonorrhea		
	High v No FQHCs	26.5 (12.2, 40.9) ***<br>Model diagnostics: F stat p-value < 0.001, adjusted R^2 = 0.190, mean residual = 0.
	Low v No FQHCs	51.9 (36.2, 67.6) ***<br>Model diagnostics: F stat p-value < 0.001, adjusted R^2 = 0.285, mean residual = 0.

** $p < 0.01$; *** $p < 0.001$.
